# Buckling Analysis of Extruded Polystyrene Columns with Various Slenderness Ratios

**DOI:** 10.3390/polym17222997

**Published:** 2025-11-11

**Authors:** Hiroshi Yoshihara, Koki Yoshimura, Masahiro Yoshinobu, Makoto Maruta

**Affiliations:** 1 Faculty of Science and Engineering, Shimane University, Nishikawazu-cho 1060, Matsue 690-8504, Japan; yoshinura8982@gmail.com (K.Y.); yosinobu@riko.shimane-u.ac.jp (M.Y.); 2 Faculty of Science and Technology, Shizuoka Institute of Science and Technology, Toyosawa 2200-2, Fukuroi 437-8555, Japan; maruta.makoto@sist.ac.jp

**Keywords:** extruded polystyrene, buckling test, buckling stress, slenderness ratio, compression test, three-point bending test

## Abstract

Extruded polystyrene (XPS) has recently been used for construction such as in walls, and floors. When it is used for walls, axial load is inevitably applied along the length direction, raising concerns of collapse owing to buckling deformation. To address this, the buckling behavior of XPS should be appropriately characterized. However, such characterization has often been ignored because XPS has not conventionally been used as a structural material but solely as a thermal insulation material. In addition, the classical methods typically applied to analyze buckling behaviors are well-established; therefore, many researchers might not consider buckling analysis to be novel. However, as the use of XPS in construction increases, its buckling behaviors cannot be ignored, and few studies have investigated them to date. In this study, buckling tests of XPS were conducted using columns with various slenderness ratios, and the buckling stress–slenderness ratio was analyzed using the following three methods: the authors’ proposed method, Southwell’s method, and the modified Euler method. Independently of the buckling tests, short column compression and three-point bending tests were performed, and the buckling stress–slenderness ratio relationship was predicted using the properties obtained from these tests. Buckling stress could be effectively determined by these three methods across a wide range of slenderness ratios, whether elastic or inelastic buckling has occurred. Our proposed method was superior to the other two methods owing to its simplicity. In contrast, it was difficult to predict the buckling stress–slenderness ratio using the properties obtained from either the compression tests alone or three-point bending tests alone. However, the relationship could be appropriately determined using the properties obtained from both tests together.

## 1. Introduction

Foamed plastics have been conventionally used as heat-insulating materials and cushioning materials, owing to their high thermal insulation and lightweight properties, respectively [1,2]. In addition, they have found use in construction for floors and walls in housing because they are free from formaldehyde, a cause of sick building syndrome, as well as efficiently attenuating seismic forces [3,4,5,6,7,8,9,10,11,12,13,14,15,16,17,18,19]. As the use of foamed plastics in construction increases, it is essential to gain a deeper insight into their mechanical properties. In particular, when they are used for walls, axial load is inevitably applied along the length direction, raising concerns of collapse owing to buckling deformation. To address this, it is important to characterize the buckling properties of foamed plastics.

Foamed plastics are often used in energy-absorbing structures under compressive loading [20,21,22,23]; therefore, compression tests of foamed plastics often performed. In contrast, although several studies have investigated the buckling properties of composite materials where foamed plastics were used as a sandwich core [24,25,26], but few have explored the buckling properties of the foamed plastics themselves [27,28,29,30,31]. Their buckling behaviors have often been ignored because they have not conventionally been used as structural materials but rather as thermal insulation materials, as described above. In fact, it is difficult to find characterization methods in major standards, including the International Organization for Standardization (ISO), ASTM International, and Japanese Industrial Standards (JIS). Although a method for detecting buckling load is standardized for continuous ceramic matrix composites in ISO 20504:2006 [32] and JIS R 1673:2007 [33], the principal aim of these standards is the prevention of buckling. This lack of standardization for foamed plastics specifically makes characterizing their buckling properties more difficult. In addition, the methods typically applied to analyze buckling behavior are well-established; therefore, many researchers might not consider buckling analysis to be novel. However, as described above, buckling behaviors cannot be ignored, as the frequency of using foamed plastics as construction materials is increasing. When foamed plastics are used for walls, it is important to analyze plate buckling behaviors, which are more complex than column buckling behaviors, but even the latter remain insufficiently elucidated.

The buckling properties of columns are usually characterized by the buckling stress (or buckling load) corresponding to the slenderness ratio. In this characterization, actual buckling tests are performed and the relationship between the load and loading-line displacement or that between load and lateral deflection is often used [34,35,36,37]. Otherwise, the strains of the column are measured on both side surfaces, and the buckling is determined when the strain reversal is induced in one surface [38,39]. However, it is often difficult to determine buckling stress precisely using these methods, as detailed below. In previous studies, the authors proposed a method for determining buckling stress using solid wood and cardboard samples with a high slenderness ratio [40,41,42]. This method is promising for determining the buckling stress of foamed plastics while reducing the drawbacks of the conventional methods described above.

Instead of performing buckling tests, buckling stress can be predicted using the stress–strain relationship obtained from compression tests of short columns [43]. In this method, when the slenderness ratio is sufficiently large, the buckling stress is predicted based on classical Euler theory. In contrast, when the slenderness ratio is regarded to be intermediate, the buckling stress is predicted using Engesser–Kármán theory or other empirical formulas [34]. However, the applicability of these methods for foamed plastics, including extruded polystyrene (XPS), has not been well verified.

In this study, buckling tests were performed using XPS columns with various slenderness ratios, ranging from intermediate to long, and the buckling stress was analyzed using three different methods. In addition, compression and three-point bending tests were performed independently of the buckling tests, and the data obtained from them were also used to predict buckling stress. As mentioned earlier, the behaviors of column buckling of foamed plastics have been scarcely investigated and remain poorly revealed. However, as the analyses of column buckling advance, they evolve into the analyses of plate buckling, which are more complex but crucial for practical applications of foamed plastics in walls and floors. Therefore, the establishment of the analysis method of column buckling behaviors are important, and this research marks the beginning of buckling analysis for foam plastics and is therefore significant.

## 2. Theoretical Background

### 2.1. Buckling Stress Determination

**Figure 1:**
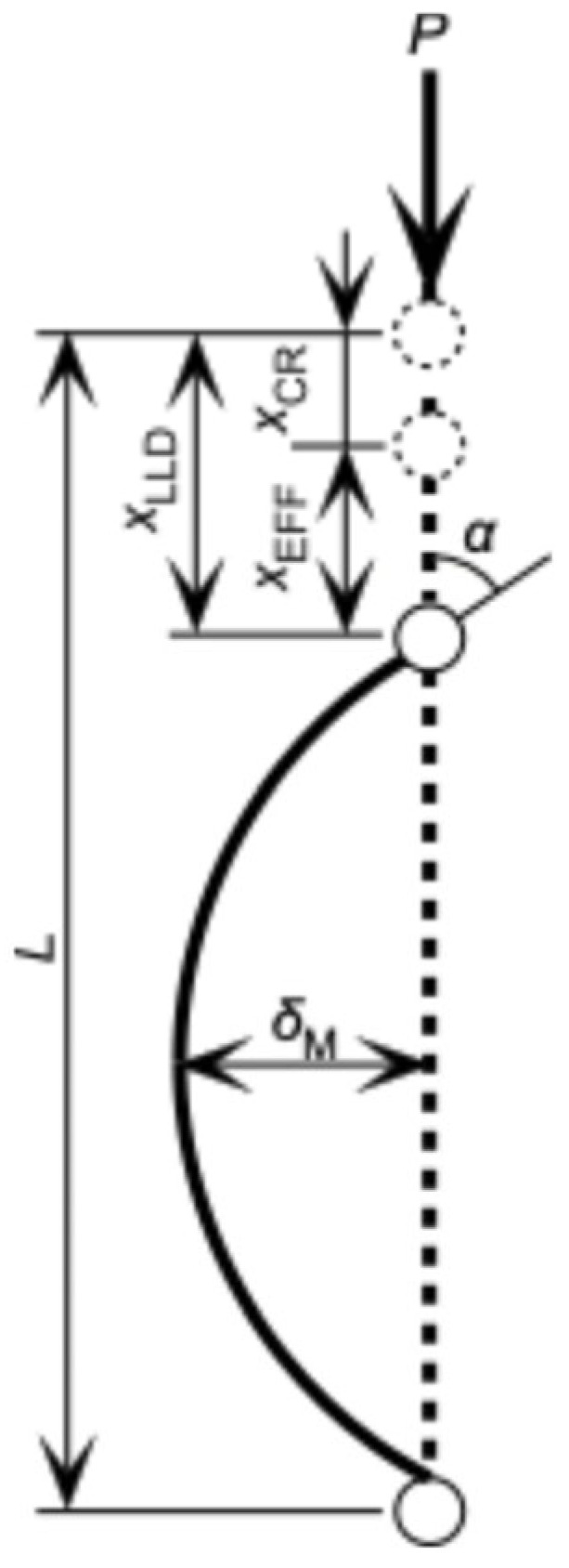
Diagram of the pre- and post-buckling conditions in an axially loaded column. Dashed and solid lines represent the pre- and post-buckling conditions, respectively.

Figure 1 represents a diagram of the column in the pre- and post-buckling conditions. The length, width, and depth of the column are defined as *L*, *B*, and *H*, respectively, whereas the axial load applied to the column and loading-line displacement are defined as *P* and *x*_LLD_, respectively. Based on these definitions, the axial stress induced in the column *σ*_AX_ is derived as *P*/(*BH*). Buckling is induced when *σ*_AX_ reaches the critical stress for buckling (i.e., buckling stress) *σ*_CR_, whereas *x*_LLD_ at *σ*_AX_ = *σ*_CR_ is defined as *x*_CR_

**Figure 2:**
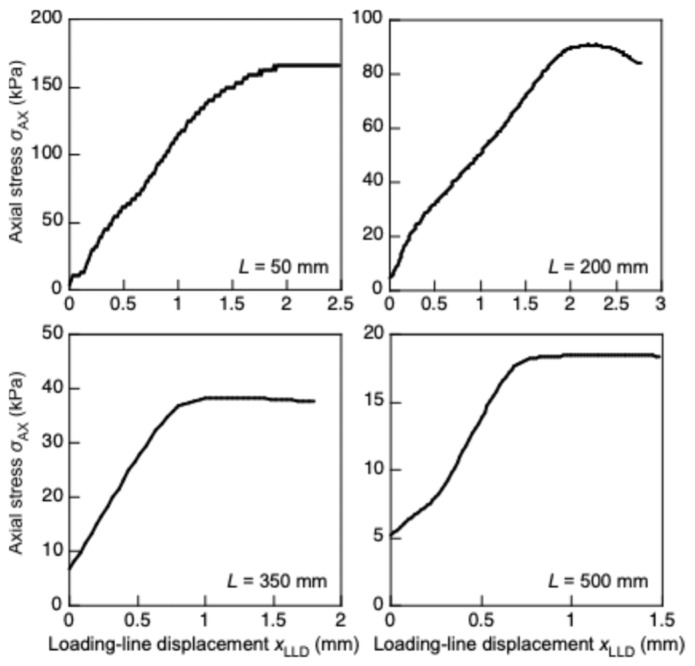
*σ*_AX_–*x*_LLD_ obtained using XPS samples with various lengths.

Figure 2 shows the typical *σ*_AX_–*x*_LLD_ relationships obtained under various *L* in this study, with these relationships including significantly nonlinear and plateau portions. In this experiment, flexural deformation was not visually observed in the linear portion of the relationship. Therefore, buckling was supposed to be induced at a certain load in the post-linear portion. Several methods have been suggested to determine the *σ*_CR_ value using the *σ*_AX_–*x*_LLD_ relationship. Kúdela and Slaninka [36] and Kotšmíd and Beňo [38] determined buckling stress using the stress at the proportional limit in the *σ*_AX_–*x*_LLD_ relationship. In their method, a straight-line is drawn onto the linear segment in the *σ*_AX_–*x*_LLD_ curve. Based on visual observation, the deviation point between the straight-line and *σ*_AX_–*x*_LLD_ curve is determined to be *σ*_CR_. However, this determination is often prone to error owing to the subjectivity in the visual observation. In contrast, Fairker [35] and Koczan and Kozakiewicz [37] determined buckling stress as the maximum of *σ*_AX_. However, in the buckling test, flexural deformation is often induced prior to the maximum *σ*_AX_. Instead of using the *σ*_AX_–*x*_LLD_ relationship, buckling stress can be determined from the strains obtained using strain gauges bonded on both surfaces of the sample [38,39]. In the pre-buckling condition, compressive strains are induced on both surfaces. After flexural deformation is induced, the polarity of the strain at the convex surface inverts, and *σ*_CR_ is determined from *σ*_AX_ at the occurrence of the strain inversion. However, this method is not always suitable for porous materials such as XPS because the strain cannot be precisely measured using strain gauges [44]. Due to the aforementioned drawbacks, these conventional methods are not always relevant for determining the *σ*_CR_ value of XPS.

There are several methods of making it easier to determine *σ*_CR_ using the load–deflection relationship in the post-buckling condition. As shown in Figure 1, the deflection at the mid-length of the sample is defined as *δ*_M_, whereas the angle between the loading line and sample length is defined as *α*. The radius of curvature at the mid-length is denoted as *κ*_M_. The effective displacement for inducing the deflection is defined as *x*_EFF_ and is obtained by subtracting *x*_CR_ from *x*_LLD_, as shown in Figure 1. According to elastica theory, *α* = 0–30°, 30–60°, and 60–90° correspond to *x*_EFF_*/L* = 0–0.0676, 0.0676–0.259, and 0.259–0.543, respectively, and the *δ*_M_*/L*–*x*_EFF_*/L* and *κ*_M_*L*–*x*_EFF_*/L* relationships under a pin–pin end condition are approximated as follows [40,41,42]:

(1) $$\frac{\delta_{M}}{L}=\left\{\begin{array}{c} 0.624{\left(\frac{x_{EFF}}{L}\right)}^{0.497}\;\;\;\;\;\;\;\;\left(0\leq x/L\leq 0.0676\right)\\ 0.549{\left(\frac{x_{EFF}}{L}\right)}^{0.450}\;\left(0.0676<x/L\leq 0.259\right)\\ 0.473{\left(\frac{x_{EFF}}{L}\right)}^{0.340}\;\left(0.259<x/L\leq 0.543\right) \end{array}\right.$$

and

(2) $$\kappa_{M}L=\left\{\begin{array}{c} 6.36{\left(\frac{x_{EFF}}{L}\right)}^{0.502}\;\;\;\;\;\;\;\;\left(0\leq x/L\leq 0.0676\right)\\ 6.87{\left(\frac{x_{EFF}}{L}\right)}^{0.530}\;\left(0.0676<x/L\leq 0.259\right)\\ 7.51{\left(\frac{x_{EFF}}{L}\right)}^{0.596}\;\;\;\left(0.259<x/L\leq 0.543\right) \end{array}\right.$$

The flexural stress and longitudinal strain at the mid-length surface, *σ*_FLEX_ and *ε*_FLEX_, respectively, are derived as

(3) $$\sigma_{FLEX}=\frac{6P\delta_{M}}{BH^{2}}$$

and

(4) $$\varepsilon_{FLEX}=\frac{\kappa_{M}H}{2}$$

**Figure 3:**
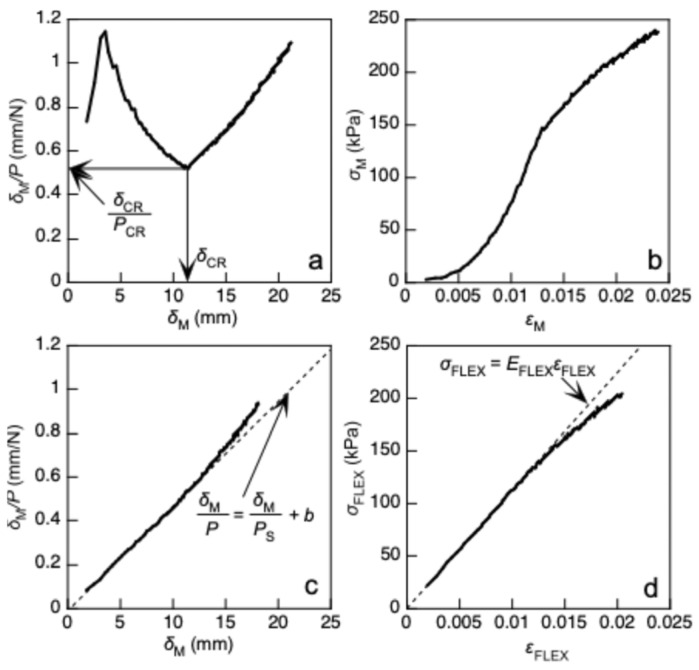
(**a**,**b**) *δ*_M_/*P*–*δ*_M_ and *σ*_FLEX_–*ε*_FLEX_ relationships obtained directly by substituting *x*_LLD_ into *x* in Equations (1) and (2), respectively, and (**c**,**d**) *δ*_M_/*P*–*δ*_M_ and *σ*_FLEX_–*ε*_FLEX_ relationships obtained by substituting the loading-line displacement after subtracting *x*_CR_ from *x*_LLD_, respectively.

Figure 3a,b show the *δ*_M_/*P*–*δ*_M_ and *σ*_FLEX_–*ε*_FLEX_ relationships obtained directly by substituting *x*_LLD_ into *x*_EFF_ in Equations (1) and (2), respectively; both relationships showed anomalous tendencies. In a previous study, *x*_CR_ was determined by deriving the minimum value of *δ*_M_/*P*, as shown in Figure 3a [40,41,42]. The *P* and *δ*_M_ values at *x*_LLD_ = *x*_CR_ are denoted as *P*_CR_ and *δ*_CR_, respectively. Figure 3c,d illustrate the *δ*_M_/*P*–*δ*_M_ and *σ*_FLEX_–*ε*_FLEX_ relationships obtained by substituting *x*_EFF_ (=*x*_LLD_ − *x*_CR_) into Equations (1) and (2), respectively. The anomalous tendencies found in Figure 3a,b are effectively reduced. Using this method, *σ*_CR_ is derived as *P*_CR_/(*BH*). Hereafter, this method is denoted as Method (A).

When using the buckling test data, the *σ*_CR_ value can be evaluated using the following two classical methods in addition to Method (A):

(B) Southwell’s method:

In Southwell’s method, the buckling load is determined using the *δ*_M_/*P*–*δ*_M_ relationship. As represented in Figure 3c, the *δ*_M_/*P*–*δ*_M_ relationship is linear at the initiation of flexural deformation; therefore, it can be regressed into the following equation [43]:

(5) $$\frac{\delta_{M}}{P}=\frac{\delta_{M}}{P_{S}}+b$$

where 1/*P*_S_ and *b* correspond to the slope and intercept of the regressed relationship, respectively; therefore, *P*_CR_ is obtained from the inverse of the slope (=*P*_S_). Similarly to Method (A), *σ*_CR_ is derived as *P*_CR_/(*BH*).

(C) Modified Euler method:

According to classical Euler theory, *σ*_CR_ is predicted using data obtained from compression tests using a short column, which is performed independently of the buckling test, as follows [43]:

(6) $$\sigma_{CR}=\frac{\pi^{2}E_{COMP}}{\lambda^{2}}$$

where *E*_COMP_ is the Young’s modulus determined using the compression test, and *λ* is the slenderness ratio. For a sample with a rectangular cross section, *λ* is derived using the cross–sectional area *A* (=*BH*) and secondary moment of area *I* (=*BH*^3^/12) as follows:

(7) $$\lambda =\frac{L}{\sqrt{\frac{I}{A}}}=\frac{2\sqrt{3}L}{H}$$

As shown in Figure 3d, the initial slope of the *σ*_FLEX_–*ε*_FLEX_ relationship is denoted as *E*_FLEX_, and *σ*_CR_ is derived using *E*_FLEX_ instead of *E*_COMP_ in Equation (6) as follows:

(8) $$\sigma_{CR}=\frac{\pi^{2}E_{FLEX}}{\lambda^{2}}$$

### 2.2. Buckling Stress Predicted from the Compression Test Using Short Column

As described above, *σ*_CR_ can be predicted using *E*_COMP_ obtained from the compression tests of short columns based on classical Euler theory. Although classical Euler theory is applicable alone for a slender column where the buckling is induced prior to the onset of nonlinearity, it was extended to Engesser–Kármán theory to predict the buckling stress of a column across a wide range of slenderness ratio. To predict *σ*_CR_ according to Engesser–Kármán theory, the stress–strain relationship obtained using compression tests should be formulated into an equation. The relationship between the compression stress *σ*_COMP_ and compression strain *ε*_COMP_ is approximated into the following Ramberg–Osgood type equation [45]:

(9) $$\varepsilon_{COMP}=\frac{\sigma_{COMP}}{E_{COMP}}+K_{COMP}{\left(\frac{\sigma_{COMP}}{F_{COMP}}\right)}^{n_{COMP}}$$

where *F*_COMP_ is the compressive strength, and *K*_COMP_ and *n*_COMP_ are the parameters obtained by regression. Using Equation (9), the tangent modulus, denoted as *E*_TAN_, is derived as follows:

(10) $$E_{TAN}=\frac{d\sigma_{COMP}}{{d\varepsilon }_{COMP}}=\frac{1}{\frac{1}{E_{COMP}}+n_{COMP}K_{COMP}{\left(\frac{\sigma_{COMP}}{F_{COMP}}\right)}^{n_{COMP}-1}}$$

According to Engesser–Kármán theory, *σ*_CR_ is represented using *E*_TAN_ instead of *E*_COMP_ in Equation (6) as follows:

(11) $$\sigma_{CR}=\frac{\pi^{2}E_{TAN}}{\lambda^{2}}=\frac{\pi^{2}}{\lambda^{2}\left[\frac{1}{E_{COMP}}+n_{COMP}K_{COMP}{\left(\frac{\sigma_{CR}}{F_{COMP}}\right)}^{n_{COMP}-1}\right]}$$

The *σ*_CR_ value can be obtained by solving Equation (11) numerically. This method is defined as Method (D).

Method (D) is not always convenient because of the complexity in solving Equation (11). Several methods have been proposed to address this, such as the Johnson–Euler method, in which the *σ*_CR_ is derived using the following two formulas [46]:

(12) $$\sigma_{CR}=\left\{\begin{array}{c} F_{COMP}-\frac{1}{E_{COMP}}{\left(\frac{F_{COMP}}{2\pi }\right)}^{2}\lambda^{2}\;\;\left(0≦\lambda ≦\lambda_{y}\right)\\ \frac{\pi^{2}E_{COMP}}{\lambda^{2}}\left(\lambda ≧\lambda_{y}\right) \end{array}\right.$$

where *λ*_y_ is the slenderness ratio at the intersectional point between the two formulas in Equation (12); therefore,

(13) $$F_{COMP}-\frac{1}{E_{COMP}}{\left(\frac{F_{COMP}}{2\pi }\right)}^{2}\lambda_{y}^{2}=\frac{\pi^{2}E_{COMP}}{\lambda_{y}^{2}}$$

The method for determining the *σ*_CR_–*λ* relationship using Equation (12) is defined as Method (E).

## 3. Materials and Methods

### 3.1. Materials

An XPS panel (STYROFOAM IB; Dupont Styro Corporation, Tokyo, Japan) was used for the tests. It had initial dimensions of 1820, 910, and 25 mm in length, width, and thickness, respectively, the directions of which are denoted as the L-, T-, and Z-axes, respectively. The panel was roughly cut using a heat cutter into smaller dimensions, and the final dimensions of the sample were cut using a heat wire. The density of the sample was 28.7 ± 0.4 kg/m^3^. The length direction of the sample coincided with the L- and T-axes of the panel. The former and latter samples were defined as L- and T-type samples, respectively. In both types, the width direction coincided with the *Z*-axis. Five samples were used for each test condition. A universal testing machine (AUTOGRAPH AG-100kNG, Shimadzu Corporation, Kyoto, Japan, capacity = 100 kN, crosshead speed accuracy = ± 0.1% of test speed) was used for all the tests. Before and during the tests, the samples were conditioned at a constant temperature of 20 °C and relative humidity of 65%. These conditions were maintained throughout the duration of the tests. The testing machine and load cells were well calibrated prior to the tests according to JIS B 7721: 2018 [47]; therefore, the errors in the testing results were caused in fluctuations in the properties of XPS panel.

### 3.2. Buckling Tests

Buckling tests were performed using the L- and T-type samples with various slenderness ratios. The value of *L* varied from 50 mm to 500 mm at intervals of 50 mm, whereas the values of *B* and *H* were fixed at 25 and 20 mm, respectively. From Equation (7), *λ* varied from approximately 8.66 to 86.6 at intervals of 8.66.

**Figure 4:**
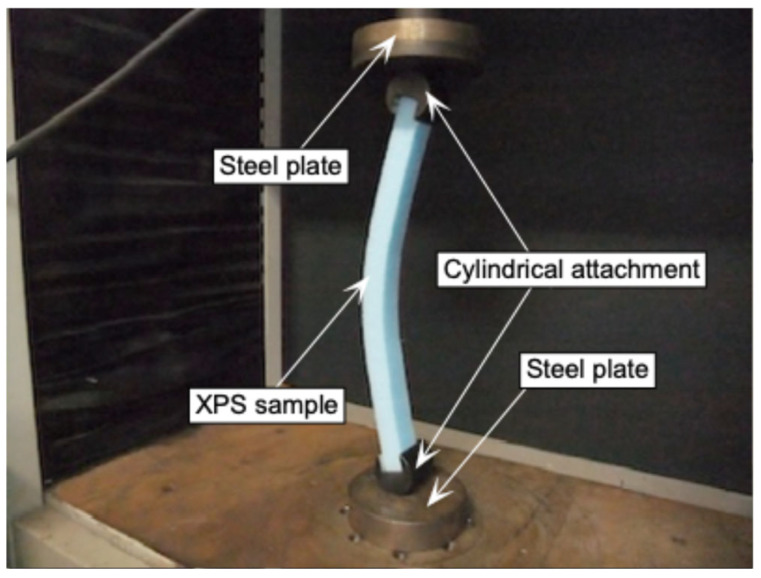
Setup for the buckling test and flexural deformation of the sample in the post-buckling condition.

Figure 4 shows a photograph of the buckling test performed in this study. An axial load *P* was applied via the cylindrical attachment equipped on both ends of the sample to realize the pin–pin end condition. The test was continued after the flexural deformation was significantly induced as shown in Figure 4. The load was measured using load cells SBL-1kN (Shimadzu Corporation, Kyoto, Japan, capacity = 1 kN, accuracy = ±10 N) and SBL-50N (Shimadzu Corporation, Kyoto, Japan, capacity = 50 N, accuracy = ±0.5 N) for the samples with *L* ranged from 50 to 150 mm and from 200 to 500 mm, respectively.

The rate of loading-line displacement (=crosshead speed) is denoted as ${\dot{x}}_{LLD}$. When *L* was greater than 250 mm, ${\dot{x}}_{LLD}$ was determined from the rate of flexural strain in the post-buckling condition ${\dot{\varepsilon }}_{FLEX}$. When *x*_EFF_*/L* is in the range of 0 to 0.0676, ${\dot{x}}_{EFF}$ is obtained using Equations (2) and (4) as follows:

(14) $${\dot{x}}_{EFF}=L{\left(\frac{0.314{\dot{\varepsilon }}_{FLEX}L}{H}\right)}^{1.99}\;\;\;\;\;\;\;\;\left(0\leq x/L\leq 0.0676\right)$$

Because *x*_LLD_ is derived by summing *x*_CR_ and *x*_EFF_, ${\dot{x}}_{EFF}$ is equal to ${\dot{x}}_{LLD}$. Therefore, ${\dot{x}}_{LLD}$ was derived by substituting ${\dot{\varepsilon }}_{FLEX}$ = 0.015/min into Equation (14) for the samples with *L* greater than 250 mm. However, when ${\dot{x}}_{LLD}$ was determined by Equation (14) for the samples with *L* lower than 200 mm, the flexural deformation was not induced easily, and the testing time often exceeded 15 min. Therefore, the crosshead speed was fixed at 0.87 mm/min when *L* was lower than 250 mm. Table 1 lists the values of ${\dot{x}}_{LLD}$ corresponding to *L*. The total testing time was approximately 5 min under these testing conditions.

When *x*_LLD_ was directly substituted into *x*_EFF_ in Equations (1) and (2), the *δ*_M_/*P*–*δ*_M_ relationship was obtained as shown in Figure 3a. Therefore, by substituting *x*_LLD_ directly into *x*_EFF_ in Equations (1) and (2), the *x*_CR_, *δ*_CR_, and *σ*_CR_ values were determined using this *δ*_M_/*P*–*δ*_M_ relationship at the minimum of *δ*_M_/*P*, according to Method (A). Then the *σ*_CR_ values were determined according to Methods (B) and (C) based on the aforementioned procedures.

### 3.3. Compression Tests Using Cubic Samples

**Figure 5:**
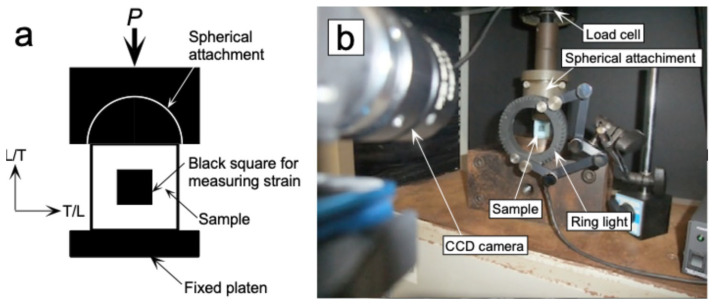
Detail (**a**) and setup (**b**) of the compression test.

Compression tests were performed using short columns (cubic samples) to obtain the *σ*_COMP_–*ε*_COMP_ relationship. Figure 5a,b show the detail and setup for the compression test, respectively. A cubic sample with dimensions of 25, 25, and 25 mm was used. As shown in Figure 5a, a black square with dimensions of 10 and 10 mm was marked using a stamp at the center of an LT-plane to measure *ε*_COMP_. A load was applied along the L- or T-direction of the sample via a spherical attachment to reduce the bending moment at both ends of the sample with a crosshead speed of 0.5 mm/min, and *σ*_COMP_ was obtained. The load was measured using a load cell SBL-1kN. During the test, the length along the loading direction of the black square was measured using a CCD camera at intervals of 0.5 s (Figure 5b), and *ε*_COMP_ was analyzed using a high-speed digital image sensor (Keyence CV-5000, Keyence Corporation, Osaka, Japan). The *σ*_COMP_–*ε*_COMP_ relationship was regressed into Equation (9), and the *E*_COMP_, *F*_COMP_, *n*_COMP_, and *K*_COMP_ values were obtained. Using these properties, the *σ*_CR_–*λ* relationships were determined according to Methods (D) and (E).

### 3.4. Three-Point Bending Tests

The *σ*_CR_–*λ* relationship could not be obtained accurately using the Engesser–Kármán and Johnson–Euler methods when using the properties obtained from the compression tests. To improve the accuracy, three-point bending tests were performed in addition to the compression tests, and the properties obtained from the former were also used to determine the *σ*_CR_–*λ* relationships using the Engesser–Kármán and Johnson–Euler methods.

**Figure 6:**
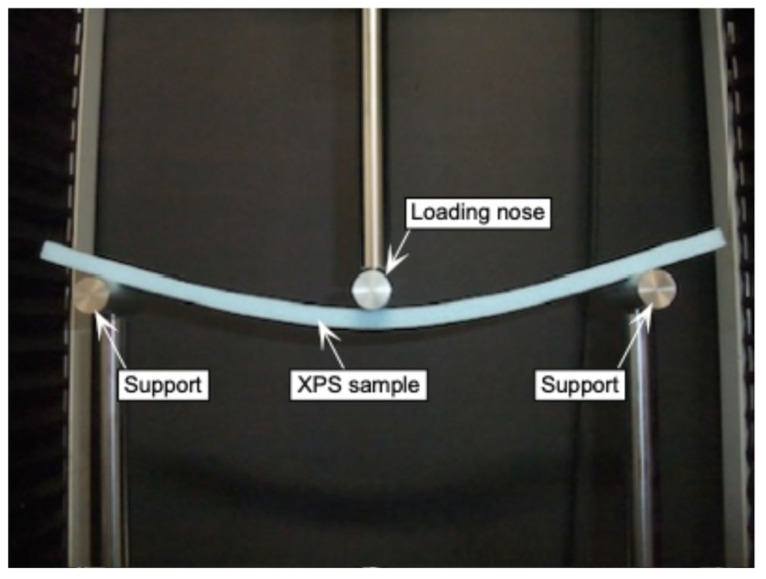
Setup for the three-point bending test.

Figure 6 shows the setup for the three-point bending test. The length, depth, and width of the sample were 400, 10, and 25 mm, respectively. Similarly to the buckling test, the length direction of the sample corresponded to the L- or T-axes. The distance between the supports *l* was 300 mm, and a load *P*_TPB_ was applied to the midspan with a crosshead speed of 50 mm/min until the load reached its maximum. The load was measured using a load cell SBL-50N. The bending stress and bending strain at the outer surface of the midspan, *σ*_TPB_ and *ε*_TPB_, respectively, are derived as follows:

(15) $$\left\{\begin{array}{c} \sigma_{TPB}=\frac{{3P}_{TPB}l}{2bh^{2}}\;\\ \varepsilon_{TPB}=\frac{6b\delta_{TPB}}{h^{2}} \end{array}\right.$$

where *b* and *h* are the width and depth of the sample, respectively, and *δ*_TPB_ is the deflection at the midspan. Similarly to Equation (9), the *σ*_TPB_–*ε*_TPB_ relationship was regressed into the following Ramberg–Osgood-type equation [41]:

(16) $$\varepsilon_{TPB}=\frac{\sigma_{TPB}}{E_{TPB}}+K_{TPB}{\left(\frac{\sigma_{TPB}}{F_{TPB}}\right)}^{n_{TPB}}$$

where *E*_TPB_ and *F*_TPB_ are the bending Young’s modulus and bending strength, respectively, and *K*_TPB_ and *n*_TPB_ are the parameters obtained by regression. The *σ*_CR_–*λ* relationship was determined based on Methods (D) using the properties obtained from the three-point bending tests as follows:

(17) $$\sigma_{CR}=\frac{\pi^{2}}{\lambda^{2}\left[\frac{1}{E_{TPB}}+n_{TPB}K_{TPB}{\left(\frac{\sigma_{TPB}}{F_{TPB}}\right)}^{n_{TPB}-1}\right]}$$

In contrast, Equations (12) and (13) were modified using *E*_TPB_ and *F*_TPB_ as follows:

(18) $$\sigma_{CR}=\left\{\begin{array}{c} F_{TPB}-\frac{1}{E_{TPB}}{\left(\frac{F_{TPB}}{2\pi }\right)}^{2}\lambda^{2}\;\;\left(0≦\lambda ≦\lambda_{y}\right)\\ \frac{\pi^{2}E_{TPB}}{\lambda^{2}}\left(\lambda ≧\lambda_{y}\right) \end{array}\right.$$

and

(19) $$F_{TPB}-\frac{1}{E_{TPB}}{\left(\frac{F_{TPB}}{2\pi }\right)}^{2}\lambda_{y}^{2}=\frac{\pi^{2}E_{TPB}}{\lambda_{y}^{2}}$$

The *σ*_CR_–*λ* relationship was also determined using Equations (17) and (18).

## 4. Results and Discussion

### 4.1. Buckling Stress Obtained from Actual Buckling Test

**Figure 7:**
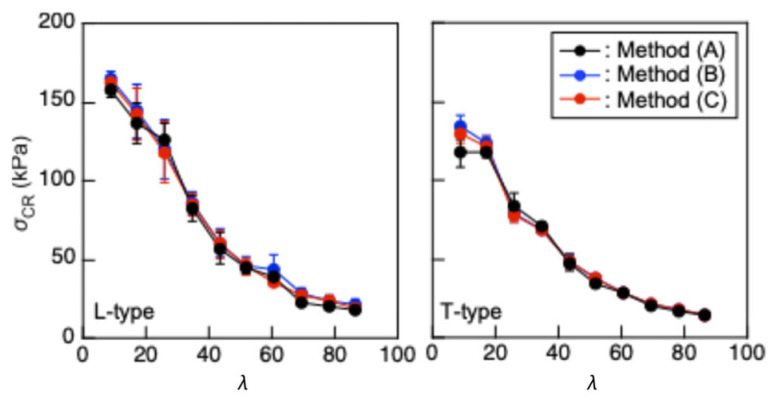
*σ*_CR_–*λ* relationship obtained using Methods (A), (B), and (C). Results = average ± standard deviations.

**Figure 8:**
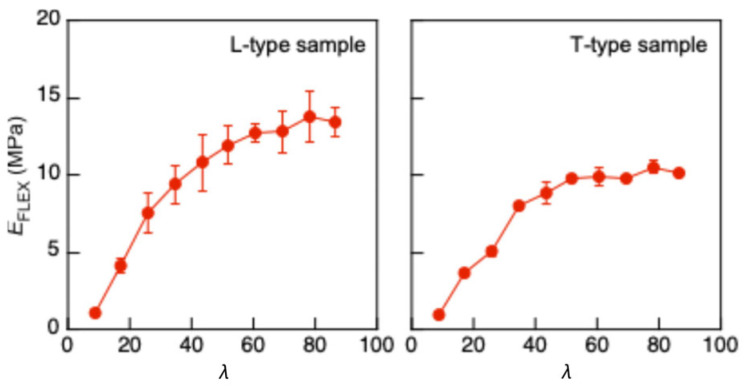
*E*_FLEX_–*λ* relationship obtained using Method (B). Results = average ± standard deviations.

Figure 7 illustrates comparisons of the *σ*_CR_–*λ* and coefficient of variation (COV)–*λ* relationships obtained using Methods (A), (B), and (C). Analysis of variance (ANOVA, Tukey tests) was performed on *σ*_CR_ corresponding to *λ* using EZR version 1.68 [48], and the *σ*_CR_–*λ* relationships obtained using the different three methods statistically coincided with each other. In a previous study, buckling analyses were performed using a slender column of solid wood and cardboard, whose *λ* exceeded 100 and 200, respectively [40,41]. There, the buckling preceded the onset of nonlinearity induced by the compressive force axially applied to the material. In contrast, *λ* was lower than 100 in this study, and the buckling was often induced after the onset of material nonlinearity. When the buckling is induced in the elastic condition, *E*_FLEX_ obtained from the *σ*_FLEX_–*ε*_FLEX_ relationship should be constant. However, as shown in Figure 8, the *E*_FLEX_ value decreased as *λ* decreased. Tukey tests were also performed, and the decreasing tendency was significant when *λ* was lower than 52.0, corresponding to *L* = 300 mm. Therefore, the buckling was induced after the material nonlinearity when *λ* was lower than 52.0, and the coincidence of the results obtained using Methods (A), (B), and (C) indicates the validity of these analysis methods. In particular, Method (A) is simpler and easier than Methods (B) and (C); therefore, it is recommended to determine the buckling stress of XPS using the actual buckling test data in a wide range of slenderness ratio.

Figure 7 also indicates that the COV values calculated using the L-type samples were often greater than those calculated using the T-type samples. The anisotropic cell arrangement in XPS may partly explain this difference, but further research involving microscopic observation is required to elucidate this phenomenon.

### 4.2. Buckling Stress Predicted Using the Compression and Three-Point Bending Test Data

**Figure 9:**
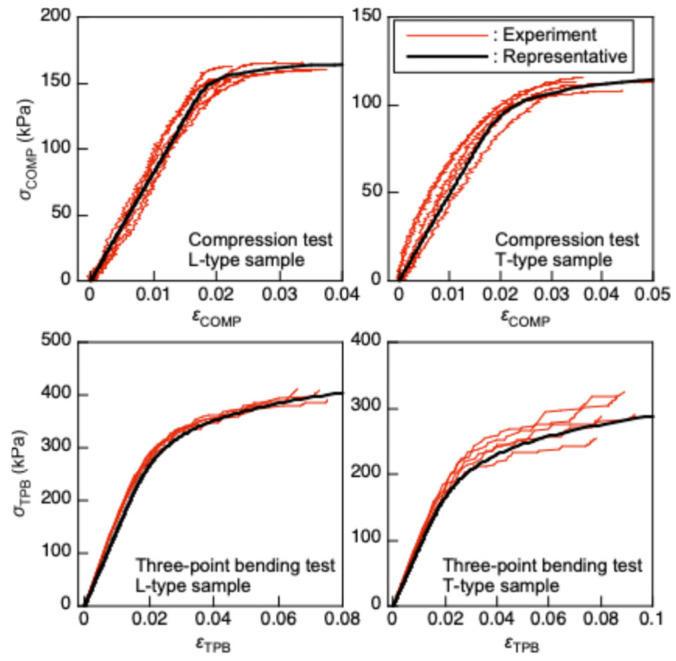
*σ*_COMP_–*ε*_COMP_ and *σ*_TPB_–*ε*_TPB_ relationships obtained via the compression and three-point bending tests, respectively.

Figure 9 illustrates the representative *σ*_COMP_–*ε*_COMP_ and *σ*_TPB_–*ε*_TPB_ relationships obtained from the compression and three-point bending tests, respectively, depicted using black solid lines. These relationships were determined as follows:

(a) The *F*_COMP_ value corresponding to each sample was determined using the maximum value of *σ*_COMP_.
(b) The experimentally obtained *σ*_COMP_–*ε*_COMP_ relationship was regressed into Equation (9), and the values of *E*_COMP_, *n*_COMP_, and *K*_COMP_ were calculated for each sample.
(c) The average value of *F*_COMP_, defined as ${\bar{F}}_{COMP}$, was calculated using five samples. Then the *ε*_COMP_ value corresponding to *σ*_COMP_ = ${N\bar{F}}_{COMP}/100$ (*N* = 1, 2, …, 100) was calculated by substituting *E*_COMP_, *n*_COMP_, and *K*_COMP_ into Equation (9).
(d) The *ε*_COMP_ values at *NF*_COMP_/100 obtained using five samples were averaged, and the averaged value was defined as ${\bar{\varepsilon }}_{COMP}$.
(e) The ${N\bar{F}}_{COMP}/100-{\bar{\varepsilon }}_{COMP}$ relationship was regressed again into Equation (9). The properties obtained from this procedure, defined as ${\bar{E}}_{COMP}$, ${\bar{n}}_{COMP}$, and ${\bar{K}}_{COMP}$, are listed in Table 2, as well as ${\bar{F}}_{COMP}$.
(f) The abovementioned process was also performed using the data obtained from the three-point tests. The values of ${\bar{E}}_{TPB}$, ${\bar{n}}_{TPB}$, ${\bar{K}}_{TPB}$ and ${\bar{F}}_{TPB}$ are also listed in Table 2.

**Table 2:** Properties calculated by regressing the stress–strain relationships obtained from the compression and three-point bending tests.

	${\bar{E}}_{COMP}$ **(MPa)**	${\bar{F}}_{COMP}$ **(kPa)**	${\bar{n}}_{COMP}$	${\bar{K}}_{COMP}$ **(×10^−3^)**
L-type	8.19	161	34.8	9.87
T-type	4.93	112	16.4	18.1
	${\bar{E}}_{TPB}$ **(MPa)**	${\bar{F}}_{TPB}$ **(kPa)**	${\bar{n}}_{TPB}$	${\bar{K}}_{TPB}$ **(×10^−3^)**
L-type	14.3	396	8.58	43.2
T-type	8.84	295	6.93	7.97

Table 2 indicates that the anisotropy of XPS affected the properties. Additionally, the properties obtained using compression and three-point bending tests are significantly different from each other. In particular, the *E*_COMP_ values are approximately half the *E*_TPB_ values, and these differences affect the prediction of the *σ*_CR_–*λ* relationship.

**Figure 10:**
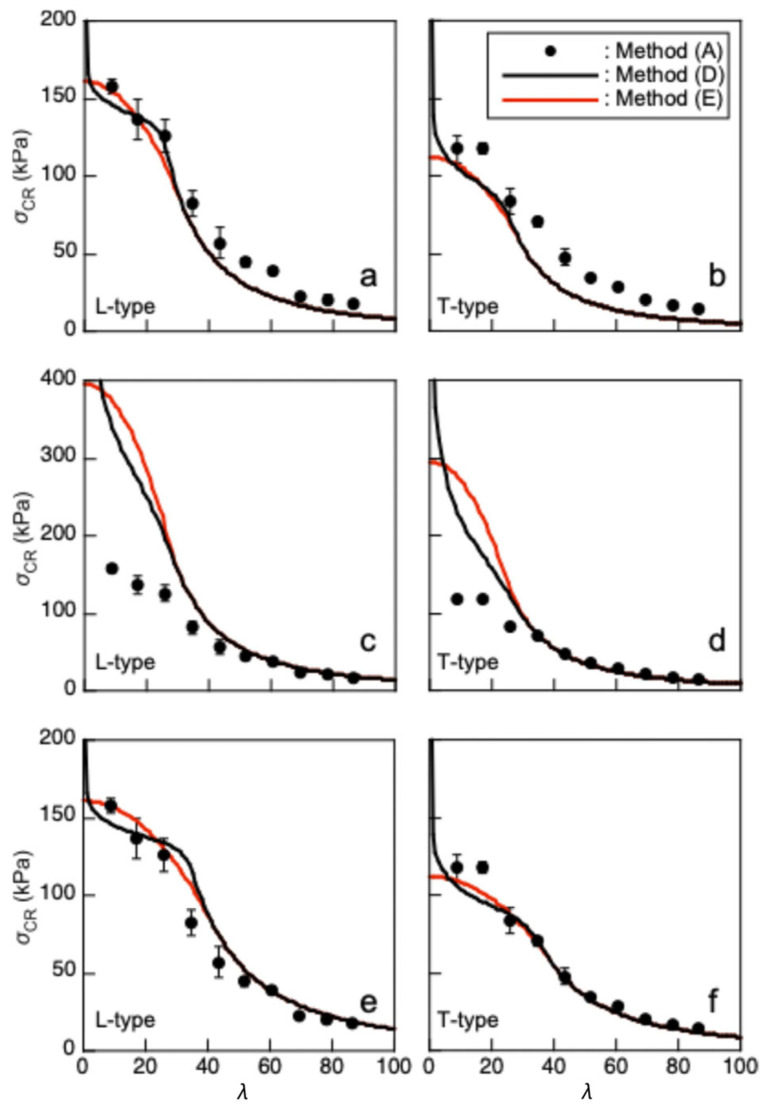
*σ*_CR_–*λ* relationships predicted using the compression and three-point bending test data. The results of Method (A) are represented as average ± standard deviations. Equations (11) and (12) are used in (**a**,**b**), Equations (17) and (18) are used in (**c**,**d**), and Equations (19) and (20) are used in (**e**,**f**).

Figure 10a,b show the *σ*_CR_–*λ* relationships predicted by Methods (D) and (E) using Equations (12) and (13), respectively. In contrast, Figure 10c,d show the relationships predicted by Equations (17) and (18). In addition to these predictions, the results obtained using Method (A), which are close to those obtained using Methods (B) and (C), are included in these figure panels. Figure 10a,b indicate that the predictions using the properties obtained from the compression tests are close to the *σ*_CR_–*λ* relationship obtained using Method (A) when *λ* is not sufficiently great. However, this closeness is not applicable as *λ* increases. In contrast, the inverse tendencies are significant when using the properties obtained from the three-point bending tests as shown in Figure 10c,d.

When *λ* is sufficiently great, the Young’s modulus is dominant in determining *σ*_CR_; therefore, the use of *E*_TPB_ is more appropriate than that of *E*_COMP_. In contrast, as *λ* decreases, the inelastic component in the stress–strain relationship becomes dominant. Considering these tendencies, Equations (12)–(14) and (17)–(19) were modified as follows:

(20) $$\sigma_{CR}=\frac{\pi^{2}}{\lambda^{2}\left[\frac{1}{E_{TPB}}+n_{COMP}K_{COMP}{\left(\frac{\sigma_{COMP}}{F_{COMP}}\right)}^{n_{COMP}-1}\right]}$$

(21) $$\sigma_{CR}=\left\{\begin{array}{c} F_{COMP}-\frac{1}{E_{TPB}}{\left(\frac{F_{COMP}}{2\pi }\right)}^{2}\lambda^{2}\;\;\left(0≦\lambda ≦\lambda_{y}\right)\\ \frac{\pi^{2}E_{TPB}}{\lambda^{2}}\left(\lambda ≧\lambda_{y}\right) \end{array}\right.$$

and

(22) $$F_{COMP}-\frac{1}{E_{TPB}}{\left(\frac{F_{TPB}}{2\pi }\right)}^{2}\lambda_{y}^{2}=\frac{\pi^{2}E_{TPB}}{\lambda_{y}^{2}}$$

Figure 10e,f represent the *σ*_CR_–*λ* relationships predicted using Equations (20) and (21). The coincidence is more significant than that when using the properties obtained from the compression or three-point bending tests alone. Buckling analyses of solid wood were performed in several previous studies, and it was found that the buckling stress of long and intermediate columns could be predicted appropriately using compression test data for a short column alone [49,50]. However, as shown in Table 2, the properties of XPS obtained using the compression and bending tests were significantly different from each other. In particular, XPS can be regarded as a bi-modular material in that the tensile and compressive Young’s moduli are different from each other. Such bi-modular characteristics are commonly found in several rocks [51]. In XPS, cell structures, including the shape and arrangement of cells, may affect the bi-modular characteristics [52]; therefore, the loading tests under microscopic observation may be effective to reveal the effects of cell structures. Further research should be conducted to reveal the bi-modular nature of XPS while performing microscopic observation. However, when both the properties obtained using compression tests and those obtained using bending tests are combined, the buckling stress of XPS can be appropriately predicted under various slenderness ratios based on Engesser–Kármán theory and the Johnson–Euler method.

Table 3 lists the values of *λ*_y_ calculated using Equations (13), (19), and (22) for L and T-type samples. For both sample types, the *λ*_y_ value increased when using the data obtained from both the compression and three-point bending tests. As described above, *λ*_y_ is the slenderness ratio at the intersectional point between the Euler’s and Johnson’s equations, corresponding to the elastic and inelastic buckling conditions, respectively. Therefore, Table 3 indicates that the inelastic buckling was induced in the large slenderness ratio greater than those predicted using the data obtained from compression or three-point bending tests alone.

From the results obtained in this study, it is recommended that the buckling stress is predicted by our proposed method when using the data obtained via the actual buckling test. Otherwise, the properties obtained from both the compression and three-point bending tests together should be used to predict the buckling stress.

As described above, foamed plastics, including XPS, are variously used in energy-absorbing structures in housing [22,23]; therefore, it is important to characterize their mechanical properties, such as buckling properties investigated in this study. However, there are several problems in the characterization of the mechanical properties. For example, it is still difficult to predict the degradation in energy-absorbing properties due to operating conditions such as creep, shock, and vibration [21]. In addition, there are still few examples examining the fracturing properties, which often dominate the strength of foamed plastics [53,54,55]. Considering that the structural use of foamed plastics will increase in the future, further research should be conducted to reveal these mechanical properties.

## 5. Conclusions

In this study, buckling tests were performed using extruded polystyrene (XPS) samples with various slenderness ratios to determine their buckling stress. In addition to the buckling tests, compression and three-point bending tests were performed independently, and the buckling stress was also predicted using the properties obtained from these tests. The dependence of the buckling test on the slenderness was analyzed, and the following results were obtained:

(1) Buckling stress could be effectively determined via the actual buckling test using our proposed method, Southwell’s method, and the modified Euler method across a wide range of slenderness ratios, whether buckling occurred in the elastic or inelastic region.
(2) Among the three methods mentioned in (1), our proposed method was superior to the other two, owing to its simplicity.
(3) It was difficult to predict the buckling stress using the properties obtained from the compression tests alone or those obtained from the bending tests alone.
(4) The buckling stress could be appropriately determined when using the properties obtained from both the compression and three-point bending tests together.

## Figures and Tables

**Table 1:** ${\dot{x}}_{LLD}$ corresponding to *L*.

*L* (mm)	50	100	150	200	250	300	350	400	450	500
${\dot{x}}_{LLD}$ (mm/min)	0.87	0.87	0.87	0.87	0.87	1.5	2.4	3.5	5.0	6.9

**Table 3:** *λ*_y_ obtained using Equations (13), (19), and (22).

Sample Type	Equation (13)	Equation (19)	Equation (22)
L-type	31.7	26.7	41.8
T-type	29.5	24.3	39.5

## Data Availability

The original contributions presented in this study are included in the article. Further inquiries can be directed to the corresponding author.

## Nomenclature

*b*	width of the sample used for the three-point bending test
*B*	width of the sample used for the buckling test
*E*_COMP_	Young’s modulus obtained from the compression test
*E*_FLEX_	Young’s modulus obtained from the buckling test under post-buckling condition
*E*_TAN_	tangent modulus
*E*_TPB_	Young’s modulus obtained from the three-point bending test
*h*	depth of the sample used for the three-point bending test
*H*	depth of the sample used for the buckling test
*l*	length between the span in the three-point bending test
*L*	length of the sample used for the buckling test
*n*_COMP_ and *K*_COMP_	parameters obtained by regressing the *σ*_COMP_–*ε*_cCOMP_ relationship into the Ramberg–Osgood type function
*n*_TPB_ and *K*_TPB_	parameters obtained by regressing the *σ*_TPB_–*ε*_TPB_ relationship into Ramberg–Osgood type function
*P*	load applied to the sample
*x*_CR_	critical displacement for buckling
*x*_EFF_	effective displacement for lateral deflection
*x*_LLD_	loading-line displacement
*ε*_COMP_	strain in the loading direction obtained from the compression test
*ε*_TPB_	strain at the surface of the midspan obtained from the three-point bending test
*λ*	slenderness ratio
*σ*_COMP_	compressive stress in the loading direction obtained from the compression test
*σ*_CR_	critical stress for buckling
*σ*_TPB_	bending stress at the surface of the midspan obtained from the three-point bending test
ANOVA	analysis of variance
COV	coefficient of variation
L, T, and Z	length, width, and thickness directions of the XPS panel, respectively
XPS	extruded polystyrene
